# Bioactives of Pomegranate By-Products and Barley Malt Grass Engage in Cereal Composite Bar to Achieve Antimycotic and Anti-Aflatoxigenic Attributes

**DOI:** 10.3390/foods11010119

**Published:** 2022-01-04

**Authors:** Ebtihal Yaqoob Khojah, Ahmed Noah Badr, Dalia Amin Mohamed, Adel Gabr Abdel-Razek

**Affiliations:** 1 Food Science and Nutrition Department, Faculty of Science, Taif University, Taif 26571, Saudi Arabia; eykhojah@tu.edu.sa (E.Y.K.); dalia@tu.edu.sa (D.A.M.); 2 Food Toxicology and Contaminants Department, National Research Centre, Dokki, Cairo 12622, Egypt; 3 Home Economic Department, Faculty of Education, Suez Canal University, Ismailia 41511, Egypt; 4 Fats and Oils Department, National Research Centre, Dokki, Cairo 12622, Egypt; adelgabr2@gmail.com

**Keywords:** aflatoxin, antimicrobial, cereal bar, barley malt grass, pomegranate byproducts, shelf-life extension

## Abstract

Food is the source from where a person obtains the body’s daily requirements. People’s current daily habits force them to consume fast food, which is known for its poor nutritional and safety features. So, it is urgent to provide a suitable substitution product to solve this issue. The present investigation aimed to produce a bar with a dual function: nutritional and long shelf life. Two materials were chosen to support the bar manufacturing regarding their bioactive contents, barley malt grass (BMG) and pomegranate byproducts (PBD). Chemical composition, antioxidant, and antimicrobial potency were measured. *Β-*carotene, vitamin C, and tocopherol were determined using HPLC apparatus. Extracts’ bio-safety against cell lines was determined, besides their enhancement against cell-death factors. Simulation experiments were designed to evaluate extracts’ impact to extend bar shelf life. Data represented the richness of essential minerals and fibers. Results of the FTIR reflected the existence of various active groups in the contents. Phenolic fractions of PBD are distinctive for their content of ellagic (39.21 ± 5.42 mg/kg), ferulic acid fractions (31.28 ± 4.07 mg/kg) which is a known with antifungal activity. Extracts and their mix (1:1) represented inhibition zone diameters that reach 15.1 ± 1.66 mm for bacteria and 23.81 ± 1.41 mm for fungi. Extracts were shown to have better safety against the cell line strain of hepatic HL-7702, with an elevation of a harmful dose of aflatoxin (IC50 304.5 µg/mL for PBD, IC50 381 µg/mL for BMG). Sensory evaluation of fortified bars reflected a preferable application of mix (1:1) due to color attributes and panelist evaluations, the same result recorded for simulation studies. The experiment recommended applying a mix (1:1) of BMG: PBD in addition to their extracts (200 mg/kg dough) for functional bar manufacturing with antifungal properties.

## 1. Introduction

Recently, the puzzle of food is related to its availability in a way that achieves food safety. However, ready-made foods suffer from food safety issues besides nutrient content troubles. Microbial contamination is the most serious challenge facing food, principally processed food [1]. Ready-to-eat foods are sources with a high content of various forms of contamination. Toxin-producing fungi play a prominent role as one of the sources of food contamination during their production from field to fork. The risk is connected to secondary metabolites secreted by these fungi that remain in food even though the fungi are eliminated by treatments [2]. Aflatoxins are reported to contaminate several foods commodities through their handling [3]. Frequently, preliminary processing of food, including heat treatments, is not sufficient to eliminate aflatoxins [4]. In addition, aflatoxins are classified as free radicals with unlimited health risks, whereas aflatoxin B_1_(AFB_1_) is classified as a pre-carcinogen. Aflatoxins’ existence in biological systems, particularly AFB_1_, leads to oxidative stress that damages tissues and causes an imbalance in some biological functions [5]. This contamination pattern of food prompted researchers to search for materials that can reduce the negative contamination impacts of food.

Plant extracts exhibited distinct substances that possess antioxidant activity and biologically active components. Those molecules overcome the effects caused by the presence of free radicals within biological systems by their antioxidant potency. Reactive oxygen species generation in healthy persons is regularly balanced by an endogenous system of antioxidative defense mechanisms. Oxidative stress is a state in which the biological equilibrium among pro-oxidants and antioxidants is disturbed to the advantage of the pro-oxidants, potentially causing harm to the body system [6]. Oxidative stress can cause molecular damage and protein carbonylation, which can contribute to several chronic illnesses [7]. Aflatoxins, as free radicals in biological systems, were shown to be influenced by phenolic substances’ existence during the in vivo and in vitro experiments. The utilization of extracts containing phenolic compounds showed a positive reduction in mycotoxin excretion.

Barley malt extract is considered an amazing source of antioxidants with in vivo and in vitro potency [8]. Previous research has shown that cereals, vegetables, and fruits are rich sources of phenolic compounds. Because of its great antioxidant contents, barley malt is getting popular as an additive in functional food manufacturing [9]. Barley malt germination is considered a beneficial method for its starch analysis that supports their enzymes activities. Malt germination was reported as a more effective extract than the regular barley extract [10]. The extract gained from barley malt after it was kilned has more enzymatic activity and achieves more beneficial characteristics for the resulting extract [11]. This kilned malting of barley may raise its antioxidant potency, which is reported to decrease oxidative stress activity [9]. Again, pomegranate byproducts are rich in their antioxidant contents. Ellagic acid and punicalagin are phenolic molecules that have peel shave antimicrobial activity [12]. The extract sourced from the peel is reported to have activity as fungicidal potency, partially against Aspergillus and Penicillium fungi [13]. Consumption of antioxidants could be a significant method for blocking or delaying the oxidation of vulnerable cellular substrates and is thus relevant to disease prevention. Recent research identified a connection between the quantity of intake of natural antioxidants sourced from food and the avoidance of several cancers [14]. Flavonoids, phenolic acids, terpenes, and tannins are examples of bioactive components that have garnered attention due to their considerable antioxidative activity [15].

A new type of food, which has the same features as fast foods and supports meal nutritional features is required. Extracts could provide a significant source for bioactive molecules with safety and nutritional properties. The formulation of composite extracts that possess high potency against aflatoxins is a novel idea. The current study focused on evaluating a composite extract formed from pomegranate byproduct (PBD) and malt barley grass (BMG) to be applied in functional cereal bar production. These extracts support the nutritional features for producing a bar, besides its influence on safety characteristics against mycotoxin contamination. One more point, this bar will enrich the biological system with biomolecules that possess antioxidant potency against in vivo free radicals.

## 2. Materials and Methods

### 2.1. Materials, Chemicals, and Micro-Organisms

The antibacterial experiment was conducted against 4 Gram-positive bacteria, *Bacillus cereus* EMCC 1080, *Streptomyces avermitilis* ATCC 31267, *Micrococcus luteus* ATCC 15176, and *Staphylococcus aureus* ATCC 13565, and also against 4 Gram-negative bacteria, *E. coli* ATCC 51659, *Salmonella typhi* ATCC 15566, *Pseudomonas aeruginosa* NRRL B-272, and *Klebsiella pneumonia* LMD 7726. These isolates were received from the DSMZ microbial collection (Leibniz Institute DSMZ-German Collection of Microorganisms and Cell Cultures, Braunschweig, Germany), maintained on nutrient agar slants for 24 h/37 °C, and kept in the refrigerator at 4 °C until use.

The antifungal experiment was done on four strains of toxigenic fungi obtained from the agro-food microbial culture collection (ITEM) microbial culture collections, ISPA, CNR, Italy. *Aspergillus flavus* ITEM 698, *Aspergillus carbonarius* ITEM 5010, *Penicillium verrucosum* NRRL 695, and *Fusarium graminearum* ATCC 56091. Before the assessment test, fungal strains were preserved on Czapek-dox media. All chemicals, standards, and media were purchased from Sigma–Aldrich Chemical Co. (St. Louis, MO, USA), where the utilized solvents and chemicals were of analytical chromatographic grade.

### 2.2. Raw Materials Extraction

The raw materials of PBD and BMG were extracted using an eco-friendly solvent system of aqueous isopropyl (80%). Milled powder was sonicated in isopropyl solution (1:4; *v*/*v*) using an Ultrasonic probe (amplitude 45%, 80 kHz, duty 60%, time 40 min, 20 °C). Extracted solutions were concentrated and dried using a lyophilized system (Laboratory Lyophilizer, FD-10-MR Malti-manifold, Esquire Biotech, Chennai, India) for utilization in further examinations.

Barley grains were purchased from the identical herbal market of a medicinal plant located in Cairo, Egypt. It was washed well before it was spread on a stainless-steel tray with a wet muslin-cloth cover. The grains were left to germinate for about 4 days, as this step increases their content of bioactive components [10], then the resultant of barley malt grass (BMG) was dried to do the kilning step [11,16] (hot air oven/40 °C/48 h). Barley grains were germinated at 22 °C for a time of 4 days, the light was valid for 12 h, and the irrigation rate was 100 mL per kg of barley. The dried matter was milled (Laboratory Mill 3310, Seedburo, Chicago, IL, USA) and vacuumed in clean sealed bags until further application. Similarly, fresh byproducts of pomegranate fruits (PBD) were collected (as a byproduct from juice processing), submerged in citric acid (2%/3 min), dried (40 °C/48 h), and milled then packed in sealed bags until further use. The average size of the particles after milling was between 0.5 and 0.7 mm.

### 2.3. Chemical Composition of Raw Materials

The Kjeldahl technique was carried out by the AOAC International procedure 981.10, according to procedures described by Mæhre et al. [17]. Roughly 1 g of raw material was hydrolyzed in a heat block digestor (420 °C/2 h) with 15 mL concentrated sulfuric acid including two copper catalyst tablets. Water was added to the hydrolysates after cooling, followed by neutralization and titration. Total protein content was calculated by multiplying the quantity of total nitrogen in the raw materials by the usual conversion ratio of 6.25 [18]. The fat analyzer (Ankom Extractor, Model XT10I O’Neil Road, Macedon, NY, USA) was utilized for the determination of fat content [19], and the crude ash content was determined after samples were ashed in a muffle furnace [20]. The AOAC enzymatic–gravimetric technique was used to calculate neutral detergent fiber (NDF), acid detergent fiber (ADF), and acid detergent lignin (ADL) [21].

The procedures of Nielsen [22] were applied to determine total carbohydrate content, and the moisture content was analyzed according to the methodology described before [23]. The mineral elements were determined using an Agilent Inductive coupled Plasma mass spectrometry (ICP-MS, 7900). The gas flow rate for nebulization was adjusted at 1 L/min, auxiliary gas had a flow rate at 1 L/min. The flow rate of Helium to the reaction cell was equal to 0.2 mL/min, and the flow rate of plasma gas was adjusted at 15 L/min. The applied detector was an electron multiplier detector (EM), Analyzer vacuum: 6 × 10^−5^, power was reported at 1500 W and 45 W for the forward and reflected power, respectively.

The phenolic fractions of the extracted materials were measured according to the methodology developed by Stuper-Szablewska et al. [24], and using the same apparatus. The phenolic content was determined at 280 and 320 nm by comparing the retention times of analyte peaks and the injected references. The quantification limit was 10 ng/g material, and the results were computed in triplicate and shown as means ± the standard error means (SEM).

### 2.4. Determination of β-Carotene, Vitamin C, and Tocopherol Contents

Carotenoids were extracted (5 g raw material) triturated using mortar and pastel with cold acetone (20 mL/3 times). The recovered solution was vacuum filtered via a Büchner funnel, transferred to cold petroleum ether (50 mL), evaporated (38 °C) by the rotary apparatus to concentrate the solution (Hei-VAP rotary evaporator, Heidolph Instruments GmbH & Co.™, Schwabach, Germany). The concentrate was dissolved in petroleum ether then kept in amber vials. Vitamin C content was extracted by the same methodology described by Campos et al. [25]. Briefly, 1 g mixed by 15 mL of extraction solution (3% meta-phosphoric acid, 8% acetic acid, 0.3 N sulfuric acid, and 1 mM EDTA), and vacuum filtrated after 5 min of mixing. The filtrate was diluted in ultrapure water (25 mL) and centrifuged (15 min/1789× *g*), then the supernatant was kept cool (4 °C). Tocopherol fractions were determined as the same methodology described before [26].

### 2.5. Determination of Antioxidant Activity

The antioxidant activities of the two types of extracts were determined using three different assays: DPPH, ABTS, and FRAP, according to the methodologies described by Abdel-Razek et al. [27]. The absorbance of the DPPH solution at 517 nm was measured using a Shimadzu spectrophotometer. In addition, the ABTS radical scavenging absorbance was taken at 734 nm using the same apparatus. The extract of BMG was evaluated by the modified method of Simic et al. [28]. Briefly, 1 mL of a 0.5 mmol/L methanol solution (of DPPH) and 2 mL of methanol were added to each extract (0.2 mL). The reaction mixture was mixed before being incubated for 30 min in the dark. The solution’s absorbance was measured against a methanol blank. Regarding the ABTS measuring activity, the ABTS compound was dissolved in water to a concentration of 7 mmol/L. The ABTS radical cation was made by mixing ABTS stock solution with 2.45 mmol/L potassium per-sulfate and storing the mixture at 22 °C for 16 h before being used. The ABTS radical cation solution was diluted using ethanol to an absorbance of 0.70 at 734 nm and equilibrated. Barley extract was combined with 2.9 mL of diluted ABTS radical cation solution in a 0.1 mL aliquot. The absorbance was measured after a 20 min reaction (at 30 °C). The inhibition of the radicals was calculated.

The FRAP assay was performed according to the methodology of [29]. The colored product’s absorbance (593 nm) was measured at zero time after vortexing. Then, samples were immersed in a water bath at 37 °C for 4 min and absorption was measured again [30]. The results were corrected for the dilution steps where the values were calculated to express the results. The antioxidant activity was measured here to indicate the biological activities of the extract, particularly as an antimicrobial and shelf-life extension.

### 2.6. Determination of Fatty Acid Contents

Fatty acid contents of the BMG, the PBD, and their mix (1:1) were determined after oil content was extracted by hexane. The composition of fatty acids was measured using the GC-apparatus using the same methodology described by Abdel-Razek et al. [31]. 

### 2.7. Determination of Bioactive Content Using the FTIR 

The Fourier-Transform Infrared Spectroscopy (FTIR) analysis was used to analyze the crude materials of BMG and PBD, as well as their mixture. The frequency range was measured as wave-numbers in the 4000–600 cm^−1^ range. In brief, the samples were put on the clean window of an Agilent Cary 630 fitted with a diamond ATR (Attenuated Total Reflectance). Micro-Lab software was utilized to evaluate the pressure clamp after it was closed until a click was heard.

### 2.8. Determination of the Antimicrobial Effect

#### 2.8.1. Antibacterial Effect

The antibacterial effect was determined using two indicators: the minimal inhibition concentration (MIC) and well assays of inhibition. The prepared extracts from the BMG, the PBD, and the mixture (1:1) were examined against bacterial strains, classified as Gram-negative bacteria and Gram-positive bacteria. The results of the MIC were determined for the tested strains according to the methodology described before [32]. The Clinical and laboratory standards institute reference methods (CLSI M7-A6) for bacteria were utilized for the broth micro-dilution examination. Concentrations of the extracts in DMSO ranged from 0.03 to 100 µg/mL. The MIC was calculated by using a microplate reader to measure each well (ASYS UVM 340, Cambridge, UK). The MIC was defined as the lowest concentration that inhibited bacterial growth as compared to the controls. Chloramphenicol (50 µg/mL) was applied as a reference antibiotic. A well-diffusion assay was applied using tested strains as referred to in a technique illustrated before [33].

#### 2.8.2. Antifungal Effect

The antifungal effect was determined using two indicators: the minimal fungicidal concentration (MFC) and the simulated growth media. The extracts of the BMG, the PBD, and the mixture (1:1) were investigated against applied strains of fungi for the two assays. The Clinical and Laboratory Standards Institute reference methods CLSI M27-A3 for fungi [32] and Itraconazole (25 µg/mL) were used as a control antifungal for the fungal strains. However, the influence of the extract was examined against the mycelia growth rate using liquid media of Czapek-Dox broth as Shehata et al. [34] illustrated before.

### 2.9. Cytotoxicity and Anti-Cytotoxic Effect of Utilized Extracts

The cell line strains of normal hepatic HL-7702 were grown at a concentration of 1 × 10^4^ cells/well (100 µL) in DMEM growth media enhanced with antibiotic treatment (0.9% saline contained 20 mg amoxicillin and 25 mg chloramphenicol), 10% phosphate saline (PBS), and incubated overnight at 37 °C and 5% CO_2_ [35]. Following the attachment, HL-7702 cells were given a serially diluted extract at dosages ranging from 1000 to 6.25 µg/mL. After that, each of the wells received 10 µL of a 12-mM MTT stock solution (5 mg/mL MTT in sterile PBS). After incubation (37 °C/4 h), the MTT solution was withdrawn. The cells’ viability was measured against the applied extracts (BMG, PBD), extracts with 200 ng AFB_1_, and AFB_1_ as a cell-death factor. The variation in the % of surviving cells versus applied concentrations was utilized to illustrate the curve by the equation outlined by Liu et al. [36].

### 2.10. Determination of Mycotoxin

The impact of the extracts was determined against the growth inhibition of toxigenic fungal strains (*A. flavus* and *F. graminarum*). Furthermore, their impact on toxin production was measured using the same methodology illustrated by Shehata et al. [34]. The mycotoxins’ amount in liquid media was determined using the Agilent 1100 (Agilent Technologies, Hewlett-Packard Strasse 876,337 Waldbronn, Germany) high-performance liquid chromatography. An Extend-C18, Zorbax column (250 mm × 5 m; 46 µm, Agilent Co., Shanghai, China) was used for the chromatographic separation. The column temperature was set to 40 °C, and the flow rate was set to 1.0 mL/min; the injection volume for samples and standard was set to 10 µL. The detector was set to excite at 360 nm and emit at 440 nm for aflatoxins, and it was set to excite at 220 nm and emit at 330 nm, respectively, for zearalenone. Data were integrated and recorded using a Hewlett-Packard Chem-Station program Manager.

### 2.11. Snack-Bar Manufacturing as a Model Application

According to the results of the initial lab trials of the components of the snack bar, which was done using several ratios of the byproducts, the snack bar was processed using 10% supplementation of the materials of PBD, BMG, and their mix (1:1) according to the confidential methodology reported by Corrigan et al. [37], with modifications. Briefly, the snack bar was prepared from raw materials containing wheat flour 220 g, 70 g glucose, 5 g sorbitol, 9 g glycerin, 56 g sunflower, 5 g soy lecithin—emulsifier, 20 g milk powder, 1.5 g salt, 13 g baking powder, 1 g vanilla, 1 g baking improver (HB Bake™—TE-Yulin, China), and 90 mL pure water. The treated dough was supported by 10% (*w*/*w*) of the planned style (BMG; PBD; a mix of them), the extracted mix was applied to all recipes except the control. As the result of the MFC for the extracted mix was recorded at 200 mg/L media, the bar improvement was done by adding 200 mg/kg dough.

### 2.12. Evaluation of the Snack-Bar Characteristics

Using a Spectro Colorimeter and the CIF lab color scale, the color parameters (L*, a*, and b*) of the control and baked bars were determined (Hunter, Lab Scan XE, Brandenburg, Germany). The L scale varies from darkness (zero) to whiteness (100); the scale stretches between the positive red Chroma to the negative green Chroma, and the scale of b spans from positive in yellow color to negative in blue color. The hardness and stickiness of the bars were measured using a Brookfield CT 3™ texture analyzer with spindles TA-TPB and TA-PFS and the accessory of the TA-BT-KIT. Utilizing the software supplied by the instrument, the data was calculated by drawing the curves. For bar testing, a 5 kg load cell was calibrated and mounted to the crosshead. The positive peak force of the graph displayed force/time versus by the texture analyzer was used to determine the hardness/compactness. The feature of a product with a low degree of hardness and a high degree of cohesion is gumminess/stickiness. Pretest speed was kept at 2 mm/s, Test speed was kept at 5 mm/s, Posttest speed was kept at 5 mm/s, and distance was kept at 30 mm on the texture analyzer (trigger force, 10 g sensitivity, 100 Load cell, 5 kg Probe).

The sensory evaluation of the snack bars was done compatibly with a reference assay (UNI, 10957: 2003) [38] through the evaluation of 50 panelists, whose age was scaled between 22 and 51, and the panel consisted of 21 men and 29 women. The panelist evaluation took place at the Food Industries and Nutrition Institute, National Research Centre (NRC). The Bars were represented in a randomized order where the panelists were asked to record their decision about the sensory attributes of the samples (appearance, color, odor, taste, texture, and overall acceptability) and give their evaluation using the 10-point hedonic scale (1 = non-preferable product, 5 = non-change product, 10 = favorite product).

### 2.13. Calorimetric Estimation of β-Glucan Content

The content of the *β-*glucan was detected after the sample was ground utilizing a mortar and pestle in liquid nitrogen. The *β-*Glucan content of bar samples was extracted using the same methodology described by Fazio et al. [39]. The *β-*Glucan content was determined using the mixed-linkage *β-*glucan kit (MegaZyme, NEOGEN, Lansing, MI 48912, USA) and the same procedures as described by McCleary and Codd [40]. The absorbance was determined using a spectrophotometer at 510 nm wavelength.

### 2.14. Simulated Experiment

The capacity of applied materials to extend the shelf life of produced bars and their resistance to contamination by the fungal strains were determined by the methodology of Badr et al. [41]. The samples were incubated at normal storage conditions (RH 92%, temperature 22 °C, normal daylight, and for 15 days storage after fungi inoculation). All the tested samples were inoculated by a strain producer of ochratoxin (*Aspergillus carbonarius* ITEM 5010). The experiment was divided into five groups: G1: negative control bars stored at 20 °C (non-inoculated); G2: positive control bars stored at 20 °C (inoculated); G3: bars manufactured using BMG; G4: bars manufactured using PBD, and G5: bars manufactured using the mix (1:1; BMG: PBD). The extension of shelf-life was recorded as the resistance to the fungi contamination on the inoculating manufactured snack bars (recorded as low fungal count of the CFU/g after the spore inoculation).

### 2.15. Statistical Analysis

All tests were performed in triplicates, and the data were reported as means ± the standard deviation. The statistical package for the social sciences (SPSS V.16) software was used to analyze the data. The analysis of variance (ANOVA) was used to assess the significant difference between the mean values, and Duncan’s multiple range test was calculated (*p* = 0.05).

## 3. Results

### 3.1. Chemical Composition of Raw Materials

Since BMG and PBD are planned to be applied in a functional snack bar with an enhanced shelf life, these materials were evaluated for their bioactive components. The data showed close values for carbohydrates and ash content (Table 1). Valuable fiber content, with a distinguished PBD content for fiber fractions, was observed. Again, the two applied materials recorded high mineral content, particularly for calcium, phosphorus, and magnesium that are known to have bio-functionality. The amount of the protein fraction appeared moderate for the raw materials; this could refer to their amino acid contents. Once more, the fiber fraction of cellulose and hemicelluloses were recorded as higher for PBD. In addition, PBD was recorded as being rich in vitamin C and vitamin E fractions (tocopherol fractions), and *β-*carotene content.

Regarding the data represented in the table of proximate analysis, significant differences between BMG and PBD content of their components were observed. While the ash content had no significant differences, protein, fats, carbohydrates, and fiber content were recorded as significant values. BMG extract showed a higher content for protein, fats, and carbohydrates, but PBD was recorded with a high fiber content. Otherwise, the fiber fraction content of BMG and PBD showed significant differences except for acid detergent lignin values. The minor element of selenium, which is known for its bio-functionality was recorded a significant value for BMG content. The content of PBD recorded significant values for vitamin C content, *β*-carotene content, and the fraction of tocopherol, while the content of *β-*tocopherol fraction was not detected.

### 3.2. Fatty Acids Composition of Raw Materials

Table 2 illustrates the individual fatty acid composition and the values of total saturated, monounsaturated, and polyunsaturated fatty acids of germinated barley, pomegranate seed oil, and the blend of BMG with PBD products. The data pointed out that the total saturated fatty acids represented 22.06, 3.70, and 7.05%, for BMG, PBD, and their mix (1:1), respectively. While the monounsaturated fatty acids (MUFA) represented 17.19, 5.27, and 6.15% for the same materials, respectively. Concerning polyunsaturated fatty acids (PUFA), which were described as the content of major fatty acids, reached 60.75, 91.03, and 86.8%, for BMG, PBD, and their mix (1:1), respectively. Such data showed that both PBD and the mix (1:1) had an increment in the PUFA content compared to BMG (91.03% and 86.8%). In this regard, the mixing process will lead to distinguished oil, which contains ω6 higher than its content for PBD and documented the highest significance for essential fatty acid (ω5, punicic acid) in the mixture compared to BMG content. Moreover, the fatty acids content of PBD and their content in the mix (1:1) contained long-chain PUFA (Omega 3, EPA) 6.31% and 2.15%, respectively. The fatty acid profile of PBD was shown containing essential linolenic acid (Omega 3) 3.59%, and it had the highest percentage of lauric, palmitic, and oleic (4.57%, 15.14%, and 14.28%) compared to the other examined samples.

It is important to note that the content of omega fatty acids in BMG was significantly higher. This means that its applications will provide significant nutritional changes in the final product. The PBD content of the distinguished ω-5 fatty acid (punicic), and its content was recorded as the dominant fatty acid of this oil. The mixing process is reflected by the support shown for some fatty acids that were recorded as not detected in the PBD content.

### 3.3. Antioxidant Activity and Active Groups of Raw Materials

**Figure 1:**
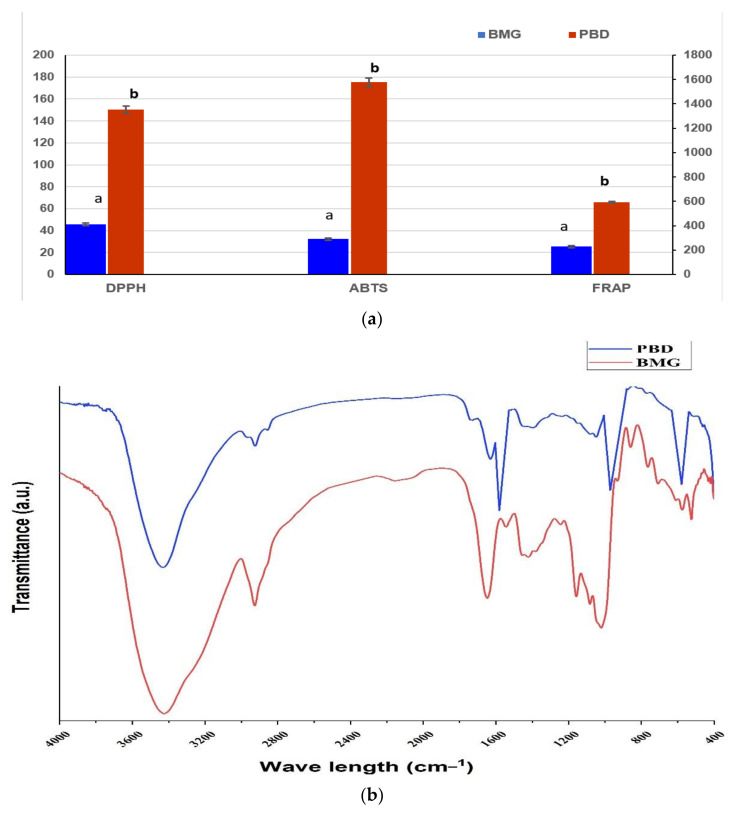
(**a**) The antioxidant activity (AA) of collected extract for BMG and PBD using three assays. (**b**) The FTIR charts diagram for BMG and PBD extracts. BMG: barley malt grass; PBD: pomegranate byproducts. AA: antioxidant activity measured as millimole Trolox equivalent per kg raw material.

The antioxidant activity of the raw material extracts, which is expected for the bar fortification, was evaluated using assays of DPPH, ABTS, and FRAP. The results reflected a high activity for the extract of PBD compared to BMG extract (Figure 1a). The results reflected significant differences between the values of BMG and the values recorded for PBD extract. The content of PBD recorded a higher antioxidant activity than BMG extract. The difference in values for each assay evaluation of each extract was recorded by major value. In this regard, the mixing process of these extracts may give a chance to support each other.

The same results were recorded for the evaluation of active groups represented in the extract (Figure 1b). The PBD curves showed more active groups than the BMG curve. While the curves of these extracts showed differences in the contents of active groups, they recorded a valuable content of *β*-D-glucan. This molecule may support the antifungal and the anti-mycotoxigenic properties in their application in food products. The represented curves also manifested a high content of hydroxyl groups, which appeared at 3200–3600 cm^−1^. These groups are known to have antimicrobial activity and their existence in the applied extract is promising. Again, the BMG curve reflected the distinguished active groups of the area of 800–1000 cm^−1^.

### 3.4. Determination of Phenolic Fractions

The phenolic compound content was determined as phenolic acids and flavonoids in the extract of BMG and PBD. The PBD contents were represented by 11 phenolic acids, and 8 flavonoid compounds compared to BMG that showed an absence of some of these components (Table 3). The two types of extracts recorded leakage of three flavonoids: namely catechol, epicatechin, and chrysin. The extract of BMG showed just three flavonoid compounds: namely catechin, apigenin, and quercetin. Regarding BMG contents of phenolic acids, ellagic, Trans-ferulic acid, and cinnamic acid were not recorded.

### 3.5. Determination of Antimicrobial Activity

The antibacterial activity of the applied extracts was evaluated against two types of pathogenic bacteria (Gram-positive and Gram-negative bacterial strains). The results of the activity were expressed in the minimal inhibition concentration (MIC), minimal bactericidal concentration (MBC), and the inhibition zone diameter (IZD) that was determined by the well-diffusion assay (Table 4). The activity was measured for BMG, PBD, and their mix (1:1) to identify the more efficient formula as an antibacterial agent. The sensitivity of the Gram-negative strains of pathogens was less than its contrary strains of the Gram-positive. Once more, the mixture extract (1:1) showed more efficient suppression of the bacterial contamination for both Gram-positive and Gram-negative strains. The strain of *Streptomyces* was recorded by a high inhibition zone diameter, while the lowest zone of inhibition was recorded for the *E. coli* strain. Generally, the application of these extracts was shown to have an antibacterial impact for the applied strains, while in some strains there were no significant differences recorded between the impact of PBD and the mixture extract. This result may connect to the high antioxidant activity of PBD that was close to the mixture of the extracts.

**Figure 2:**
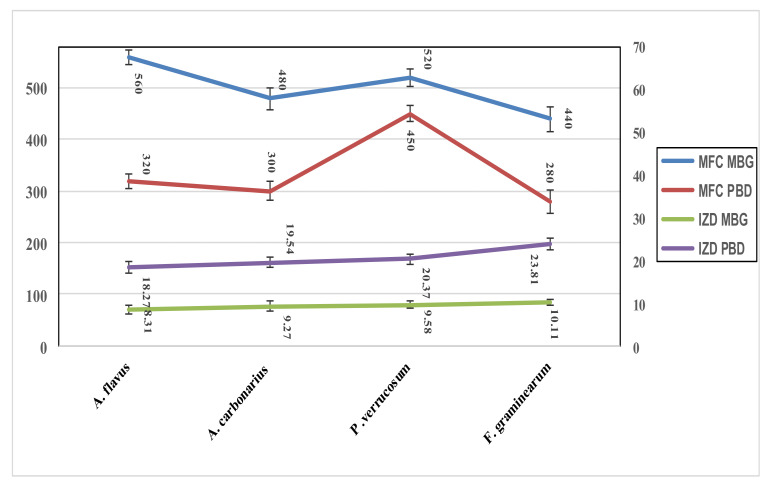
Antifungal activity of the extracts measuring as minimal fungicidal concentration and inhibition zone diameter. MFC: minimal fungicidal concentration (mg/L media); IZD: inhibition zone diameter measured in millimeter. MBG: barley malt grass; PBD: pomegranate byproduct.

However, the antifungal activity of these extracts was measured in two ways (Figure 2), estimating the minimal fungicidal concentration (MFC), which represents the amount of extract required to inhibit the fungal growth in liquid media. While the second assay that was applied to determine the antifungal activity was the well-diffusion assay, which was recorded as inhibition zone diameter which occurred due to the presence of the extract. The data represented in Figure 2, showed more activity for the extract of PBD. The higher inhibition for the two extracts was recorded against *Fusarium*.

### 3.6. Determination of Extracts Cytotoxicity

**Figure 3:**
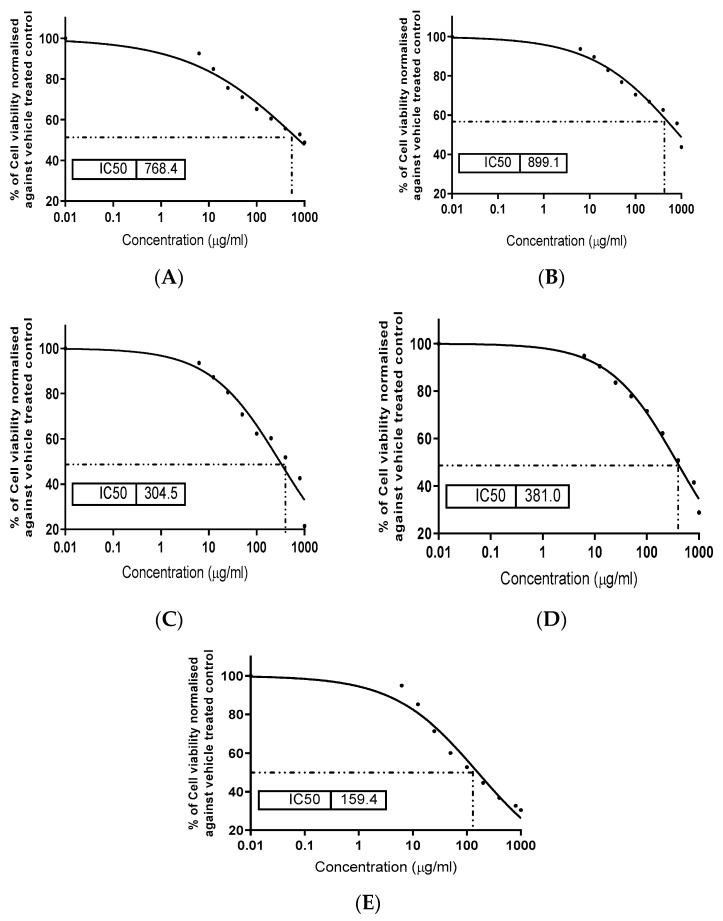
Effect of the extracts on cell line viability within/without the presence of aflatoxin B_1_. (**A**) The impact of PBD; (**B**) The impact of BMG; (**C**) The impact of PBD + AFB_1_; (**D**) The impact of BMG + AFB_1_; and (**E**) The impact of the AFB_1_ on cell line viability. MBG: barley malt grass; PBD: pomegranate byproduct; concentration was performed as µg extract/mL cell line solution.

The cytotoxic impact of the applied extracts was determined to clarify the safety of extract application. In addition, the extracts were evaluated for their anti-toxic impact against the toxicity that can be caused due to the presence of the AFB_1_ in the cell-growth media. Regarding the data represented in Figure 3, the toxicity impact of the PBD extract was higher than the impact of the BMG extract (Figure 3A,B).

However, the presence of the two types of the extract with AFB_1_ had a positive effect against the activity of the AFB_1_ activity as a cell-death factor. This effect was recorded as the existence of the extract in the cell line media contaminated by the AFB_1_ buffer solution (Figure 3A,B). The toxic effect of the AFB_1_ buffer solution was recorded clearly in Figure 3E. Compared to this figure, the PBD extract reflected an ability to save the cell as more viable (Figure 3C) followed by the BMG extract (Figure 3D) in comparison to the cell treated individually by the AFB_1_ buffer solution.

### 3.7. Determination of Extracts Role as an Anti-Mycotoxigenic Preservative

**Figure 4:**
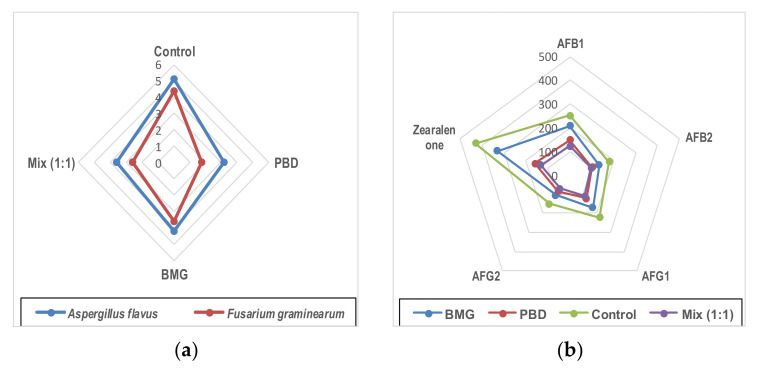
Effect of the extracts in a simulated trial against toxigenic fungi contamination and their toxins. (**a**) Represent the impact of the extracts on the fungal growth rate; (**b**) represent the impact of the extracts on toxin secretion. MBG: barley malt grass; PBD: pomegranate byproduct.

To simulate the antifungal activity of the extracts against the toxigenic fungal contamination, as well as their mycotoxin contamination due to their presence, liquid growth media was applied. The media contained two toxigenic fungal strains that are known to produce mycotoxins (*A. flavus; F. graminarum*). The strain of *A. flavus* is known to produce the four types of aflatoxins (AFB_1_; AFB_2_; AFG_1_; AFG_2_), and the strain of *F. graminarum* is known to produce zearalenone toxin. The inhibition impact for strains grown in the liquid media was recorded as more efficient for the PBD extract (Figure 4a). While the mix (1:1) recorded the highest reduction in the mycotoxin secretion effect followed by the PBD extract (Figure 4b). The close impact of the mixture extract to the impact of the PBD extract in the reduction effect in the secretion of mycotoxin from the applied fungi was noticed.

### 3.8. Characterization of Snack Bar Manufactured Targeted Materials

Color attributes, water activity, and texture analysis were determined by measuring the changes that occurred to the snack bar due to the targeted material application. The rate of change in the product color was recorded as the lowest for the bar manufactured using BMG. The changes recorded for the bar manufactured using the mix (1:1) were less than the changes that occurred via the application of PBD in bar manufacturing. The water activity of the bar samples was recorded as low values, where the control and the bar-mix were close in their values (Table 5). Regarding the hardness of the manufacturing bars, the bar-mix was the only type that was recorded as softer compared to the control. The recorded value of the stickiness for the bars manufactured using both BMG and PBD showed clear differences compared to the control, which was less noticed for the bar manufactured using the mix (1:1).

**Figure 5:**
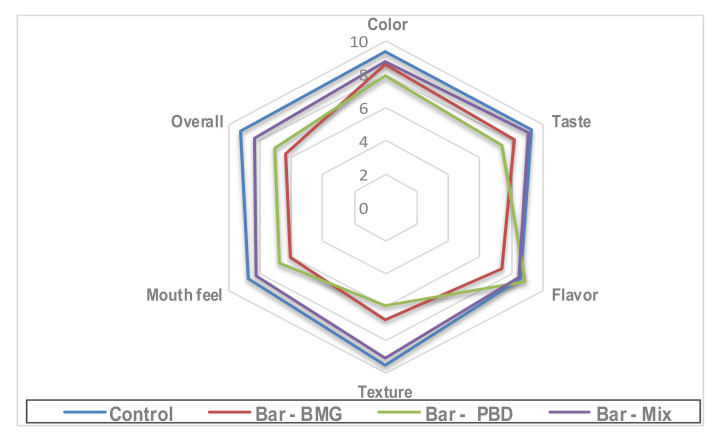
The sensory evaluation results of the bar enhanced using BMG, PBD, and their mix (1:1). MBG: barley malt grass; PBD: pomegranate byproduct. Mix: mean the addition of MBG and PBD at mixing ratio (1:1). The data of sensory evaluation for the manufactured bar is represented as means of panelist degrees.

Moreover, the analysis of the bar content of *β-*glucan content had the highest content for the bar manufactured using the mix (1:1); the value was recorded at 1.97%. The content of the bar-BMG was second (1.67%), followed by the bar-PBD (1.39%), while it was not detected for the control bar. The next step of bar evaluation was performed throughout the panelist board (Figure 5). The results reflected the compatibility of the panelist who scored close results for the taste and flavor of the control and the bar-mix. While the sensory results recorded for the bar-PBD showed a significant difference. The sensory results for texture showed high changes for the bar manufactured using individual addition of BMG or PBD for the recipe of the bar ingredients.

### 3.9. Simulated Experiment for Bar Resistance of Fungal Contamination

After the bars were inoculated with 2.1 × 10^2^ CFU of the applied fungi and stored in sealed bags, a bar was withdrawn daily for inspection and analysis. The results reflected more resistance of the bar-PBD to contamination during the storage time (Table 6). The order of bar resistance to fungal contamination was bar-PBD > bar-mix > bar-BMG > inoculated bar without treatment. The rate of contamination after the inoculation process was lower for the bar-PBD and bar-mix. After two weeks of storage at room temperature, the colony count of the inoculated bars did not exceed 10^4^ CFU of the fungi. 

## 4. Discussion

According to the fast rate of the daily routine, most humans prefer to consume the easy diets of fast meals. In this regard, the authors targeted an easily consumed type of bakery product that achieves two benefits: nutritional support and resistance to regular contamination. Accordingly, BMG and PBD were chosen to insert into a recipe for a snack bar. The dried extracts of BMG and PVBD were mixed at a ratio (1:1) and applied along with the dried powder supplementation. The evaluation of targeted materials reflected a high content of fiber, with the distinguished distribution of fiber fractions. Dietary fiber is among the key phytochemicals included in cereals that are classified into two types based on their water solubility. The water-soluble portion (soluble fiber) is mostly composed of non-starchy polysaccharides, such as *β-*glucans and arabinoxylan. In humans, soluble fiber has been shown to lower serum cholesterol, postprandial blood glucose, and insulin levels [42].

The proximate analysis of BMG and PBD reflects a good content of protein (Table 1), which translated as amino acids with health benefits, particularly for providing the body requirements of essential amino acids. The mineral content in the applied materials was shown as higher in calcium and phosphorus elements that are known to be necessary for the bone-building of the young and assist to support the elder-bone structure to avoid several health issues. PBD was rich in vitamin C, tocopherol, and carotene. These components possess bioactivity as an antioxidant and support the biological system against oxidative stress. The evaluation of antioxidant potency using three assays (Figure 1a) reflected the amazing activity, particularly for the PBD extract. Once more, the FTIR analysis showed a variation in the oligosaccharides (between 1700 and 880 cm^−1^), while the later area of the curve reflected the high content of hydroxyl groups (Figure 1b). The previous investigation reported peaks near the area of 1600–800 cm^−1^ as belonging to oligosaccharides, particularly mannan-oligosaccharides and *β-*glucan [43,44]. The application of *β-*glucan may serve to achieve bioactive functions by its existence [45]. Otherwise, the existence of such components is referred to as having activity against harmful substances, such as chelators, particularly aflatoxins [46] and zearalenone [47].

Generally, plant extracts are rich in phenolic compounds that can play a role as an antioxidant [48] and can suppress fungal contaminations to reduce mycotoxin secretion [49,50]. This activity was emphasized by several applications of plant extracts rich in phenolics, including *stevia* [51], grape-byproducts [52], moringa [53], and *opuntia* [54]. Moreover, the application of phenolic-plant extracts to experimental animal diets supports biological antioxidant enzymes and the protection against mycotoxins [55,56]. While mycotoxins can produce oxidative stress due to their chemical structure, the antioxidant molecules, phenolic compounds, may act against this action [57]. The extract of the applied materials reflected a valuable content of phenolic compounds. The PBD extract appeared richer with 11 phenolic acids and 8 flavonoids. Ellagic, cinnamic, and ferulic fractions are the phenolic acids that distinguish PBD and increase potency as an antioxidant. Quercetin and apigenin are the dominant flavonoids in PBD, while catechin was the highest flavonoid in the BMG extract.

Regarding the antimicrobial activity, two extracts manifested antibacterial activity with a preference for PBD against the applied pathogen strains. The mix of extracts (1:1) reflected a more efficient suppression of bacterial contamination (Table 4); this effect may be illustrated through the synergistic effect between minor components’ existence in them [58]. The antifungal activity of the extracts was also recorded against four toxigenic fungal strains (Figure 2), with a high inhibition zone diameter against Fusarium fungi. This activity could be illustrated by the phenolic antioxidant and bioactive molecules contents of the extracts [50]. Again, the cytotoxic effect of the extract, as well as their anti-death agent in the presence of AFB_1_ was determined using a cell line assay of a healthy strain. The results showed a safety indication for the extracts (Figure 3A,B), and an enhancement influence of cell viability when AFB_1_ existed in cell line media growth (Figure 3C,D). The impact of AFB_1_ on cell line viability as a cell-death agent is represented in Figure 3E.

**Figure 6:**
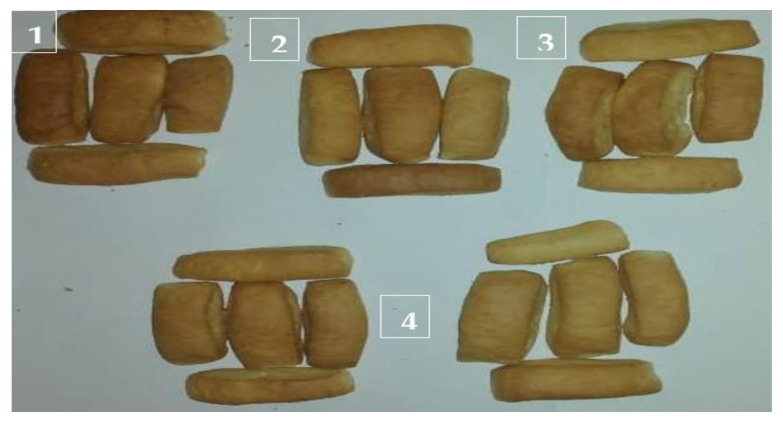
Manufactured bar of PBD, BMG, their mixture, and the control. (**1**) Composite bar with PBD; (**2**) composite bar with BMG; (**3**) composite bar with the mixture; (**4**) control bar (two groups).

The impact of the applied extracts as anti-mycotic and an anti-mycotoxigenic agent was evaluated using liquid media containing strains of toxigenic fungi. These strains are known for their ability to produce mycotoxins. The extracts were shown to be active in the suppression of the fungal growth of the applied strains (Figure 4a). In addition, their related mycotoxin secretion was shown to be reduced compared to the control strain secretion (Figure 4b). Several cereal products are known to spoil due to toxigenic fungi [41], where the application of these extracts may support their protection. The existence of *β-*glucan in the extracts may also act to chelate mycotoxins [46,47]. The application of the raw materials in addition to their extracts in the manufacture of a functional bar provides two benefits: nutritional and extending the shelf life (Table 6). The sensory evaluation of the fortified bars recommended the application of raw materials mix (1:1) within their extract at 200 mg/kg dough. The results reflected a close result for taste and aroma of the bar-mix type with the control. The simulated experiment for the shelf life referred to bar-PBD followed by bar-mix as the products with enhanced shelf life (Table 6). The overall appearance of the composite sample bar and the control bar is shown in Figure 6.

## 5. Conclusions

While fast-consumed diets are required by humans, it is vital to get a food product with unique qualities. Fast meals frequently ignore the nutritional value and pay less attention to food safety. The current study focused on a neoteric source of bioactive components that was aimed at enriching a kind of food product to accomplish nutritional, functional, and safety qualities in a simple-to-consume product. Dietary fibers provide nutritional and physiological function in BMG and PBD, which are also manifested as high in minerals and protein. Several phenolic acids and flavonoid components were found in the phenolic fractions of BMG and PBD. Aside from BMG’s valuable fatty acid composition, the antioxidant potency of PBD extract was observed by valuable effectiveness. The FTIR measurement of active groups revealed the important contents of oligosaccharides, specifically *β*-glucan. As a consequence, these materials have antibacterial and antifungal properties. The cytotoxic analysis of these materials indicated that they are safe for use in prepared foods. The combined BMG: PBD (1:1) properties exhibited additional advantages, recommending their use in cereal bars as a preferred product. The produced bar was made by combining the ingredients from BMG and PBD into a mixture of extracts. Better sensory characteristics and longer shelf life were noted in the manufactured bars. In this regard, the authors suggest using this recipe to make a functional bar that is easy to ingest and has safety qualities as well as a long shelf-life product that withstands microbial contamination.

## Figures and Tables

**Table 1:** The proximate analysis, fiber fractions, and minerals content of BMG and PBD.

**Proximate Analysis**						
	Moisture (%)	Protein (%)	Fat (%)	Ash (%)	Carbohydrates (%)	Crude fiber (%)
BMG	18.41 ± 1.34 ^a^	10.57 ± 0.84 ^a^	1.57 ± 0.37 ^a^	3.68 ± 0.87 ^a^	63.75 ± 1.27 ^a^	13.25 ± 0.41 ^a^
PBD	7.66 ± 1.23 ^b^	6.54 ± 1.05 ^b^	0.81 ± 0.05 ^b^	3.97 ± 0.67 ^a^	56.1 ± 2.14 ^b^	16.32 ± 2.73 ^b^
**Fiber fractions**						
	Cellulose	Hemi cellulose	Lignin	NDF	ADF	ADL
BMG	9.11 ± 0.63 ^e^	27.67 ± 0.36 ^e^	1.31 ± 0.11 ^e^	39.02 ± 0.54 ^e^	11.35 ± 0.22 ^e^	2.25 ± 0.77 ^e^
PBD	12.32 ± 1.63 ^f^	10.46 ± 2.13 ^f^	9.67 ± 0.41 ^f^	26.41 ± 2.18 ^f^	15.95 ± 2.54 ^f^	3.63 ± 0.84 ^e^
**Mineral content (mg/kg)**						
	Ca	Fe	P	Mg	Zn	Se
BMG	427.89 ± 1.21 ^j^	27.11 ± 0.13 ^j^	193.17 ± 1.31 ^j^	111.15 ± 1.42 ^j^	25.16 ± 0.31 ^j^	0.69 ± 0.12 ^j^
PBD	1143.2 ± 7.24 ^k^	63.15 ± 2.37 ^k^	275.14 ± 4.61 ^k^	655.37 ± 8.41 ^k^	4.77 ± 0.82 ^k^	0.11 ± 0.04 ^k^
**Tocopherol, vitamin C, and *β*-carotene contents (mg/100 g)**						
	Vitamin C	*α-*tocopherol	*β-*tocopherol	*γ-*tocopherol	*δ*-tocopherol	*β-*carotene
BMG	5.71 ± 1.17 ^r^	0.84 ± 0.74 ^r^	0.081 ± 0.05	0.54 ± 0.023 ^r^	0.93 ± 0.057 ^r^	0.17 ± 0.008 ^r^
PBD	136.3 ± 2.58 ^u^	179.92 ± 1.75 ^u^	ND	99.73 ± 0.87 ^u^	29.64 ± 0.58 ^u^	14.43 ± 0.36 ^u^

The data are expressed as means ± SD (where *n* = 3); Tocopherol fractions represent the content of vitamin E. NDF: neutral detergent fiber; ADF: acid detergent fiber; ADL: acid detergent lignin; BMG: barley malt grass; PBD: pomegranate byproduct. The values that appear with different superscript letters in the same column have significant differences. All represented fractions are considered as bioactive components, expected to have functionality for a manufactured bar.

**Table 2:** Fatty acid composition of Germinated parley oil, Pomegranate seed oil, and Mixture of them.

Fatty Acids		BMG	PBD	Mix
		(%)	(%)	(1:1)
C12:0	Lauric	4.57 ± 0.001	ND	ND
C14:1	Myristoleic	ND	ND	ND
C16:0	Palmetic	15.14 ± 0.09 ^a^	2.33 ± 0.84 ^b^	3.52 ± 0.37 ^b^
C16:1	Palmitoleic	1.74 ± 0.22 ^a^	ND	0.31 ± 0.02 ^b^
C18:0	Stearic	1.63 ± 0.005 ^a^	1.37 ± 0.54 ^b^	1.60 ± 0.41 ^a^
C18:1	Oleic	14.28 ± 0.67 ^a^	5.27 ± 0.76 ^b^	5.36 ± 0.89 ^b^
C18:2 *n*-6	linoleic	50.85 ± 1.08 ^a^	5.05 ± 0.81 ^b^	9.35 ± 1.12 ^c^
C18:3 *n*-3	linolenic	3.59 ± 0.34 ^a^	0.54 ± 0.05 ^b^	0.61 ± 0.05 ^b^
C18:3 *n*-5	punicic	ND	85.44 ± 2.23 ^a^	74.69 ± 1.63 ^a^
C20:0	Arachidic	0.72 ± 0.002 ^a^	ND	1.93 ± 0.14 ^b^
C20:1	Gadoleic	1.17 ± 0.004 ^a^	ND	0.48 ± 0.03 ^b^
C20:5 *n*-3	Eicosapentaenoic	6.31 ± 0.002 ^a^	ND	2.15 ± 0.87 ^b^
**Significant oil parameters**				
SFA		22.06 ± 3.16 ^a^	3.70 ± 1.05 ^b^	7.05 ± 1.88 ^c^
MUFA		17.19 ± 2.54 ^a^	5.27 ± 1.66 ^b^	6.15 ± 2.02 ^b^
PUFA		60.75 ± 5.02 ^a^	91.03 ± 5.73 ^b^	86.8 ± 4.79 ^b^

The data are expressed as means ± SD (where replicates = 3); *n-*: refers to omega fatty acids. SFA: saturated fatty acids; MUFA: monounsaturated fatty acids; PUFA polyunsaturated fatty acids. The values that appear with different superscript letters in the same raw have significant differences. ND: represent the values that were not detected.

**Table 3:** Phenolic compound contents of BMG and PBD extracts.

Phenolic Acids	BMG	PBD	Flavonoids	BMG	PBD
	(mg/kg)	(mg/kg)		(mg/kg)	(mg/kg)
Gallic acid	9.24 ± 1.05 ^a^	91.13 ± 2.61 ^b^	Catechin	1.05 ± 0.84 ^d^	16.94 ± 2.28 ^e^
Ellagic acid	ND	39.21 ± 5.42	Catechol	ND	ND
Protocatechuic acid	7.62 ± 1.21 ^a^	11.28 ± 1.37 ^b^	Epicatechin	ND	ND
*trans*-Ferulic acid	ND	16.17 ± 1.91	Rutin	ND	8.42± 1.08
Cinnamic acid	ND	43.16 ± 1.05	Apigenin	0.74 ± 0.16 ^d^	29.41 ± 2.27 ^e^
Syringic acid	3.08 ± 0.67 ^a^	17.39 ± 2.51 ^b^	Quercetin	0.38 ± 0.09 ^d^	28.34 ± 1.54 ^e^
Caffeic acid	4.18 ± 0.54 ^a^	18.44 ± 2.74 ^b^	Luteolin	ND	18.52 ± 1.05
Ferulic acid	15.11 ± 2.16 ^a^	8.41 ± 1.34 ^b^	Hesperidin	ND	3.55 ± 1.28
*p*-Hydroxybenzoic acid	5.13 ± 0.74 ^a^	2.88 ± 0.73 ^b^	Naringenin	ND	13.53 ± 1.44
*p*-Coumaric acid	22.61 ± 2.37 ^a^	20.08 ± 1.41 ^b^	Kaempferol	ND	4.21 ± 0.81
Vanillic acid	5.06 ± 0.33 ^a^	34.17 ± 1.02 ^b^	Chrysin	ND	ND

Data are expressed as means ± SD (where *n* = 3); mg/kg: compound quantity was measured in milligram per kg of raw material dry weight matter. ND: represent the compounds that were not detected at the detection limit. The values that appear with different superscript letters in the same row have significant differences. The amounts are represented in mg phenolic compound per kg of dry matter.

**Table 4:** Antibacterial activity of BMG, PBD extracts, and their mixture (1:1).

	MIC (mg/L)			MBC (mg/L)			IZD (mm)		
	BMG	PBD	Mix (1:1)	BMG	PBD	Mix (1:1)	BMG	PBD	Mix (1:1)
**Gram-positive bacteria**									
***Bacillus cereus*** **EMCC 1080**	180	100	80	220	150	140	7.34 ± 0.54 ^c^	12.16 ± 1.02 ^b^	14.9 ± 1.34 ^a^
***Streptomyces avermitilis*** **ATCC 31267**	170	80	50	200	150	150	7.61 ± 0.39 ^b^	13.84 ± 0.93 ^a^	15.1 ± 1.66 ^a^
***Micrococcus luteus*** **ATCC 15176**	180	90	80	220	160	150	8.12 ± 0.23 ^b^	12.55 ± 1.1 ^a^	14.6 ± 1.41 ^a^
***Staphylococcus aureus*** **ATCC 13565**	200	80	70	220	150	160	7.44 ± 0.87 ^b^	12.71 ± 1.23 ^a^	13.8 ± 1.19 ^a^
**Gram-negative bacteria**									
***Escherichia coli*** **ATCC 51659**	250	90	90	280	180	160	5.37 ± 0.97 ^c^	10.97 ± 0.94 ^b^	12.05 ± 1.05 ^a^
***Salmonella typhi*** **ATCC 15566**	250	70	70	280	200	180	5.08 ± 1.02 ^b^	11.02 ± 0.81 ^a^	12.37 ± 1.15 ^a^
***Pseudomonas aeruginosa*** **NRRL 272**	230	90	90	260	180	180	4.88 ± 0.83 ^c^	11.54 ± 0.79 ^b^	14.21 ± 1.02 ^a^
***Klebsiella pneumonia***	230	90	90	280	180	160	5.67 ± 0.67 ^b^	11.81 ± 0.88 ^a^	12.4 ± 1.21 ^a^
**LMD 7726**									

The data are expressed as means ± SD (where *n* = 3) and measured as mg extract/L media. MIC: Minimal inhibition concentration; MBC: Minimal bactericidal concentration; MBG: barley malt grass; PBD: pomegranate byproduct. The values that appear with different superscript letters in the same row have significant differences. IZD: inhibition zone diameter measured in millimeters.

**Table 5:** Color attributes, water activity, and texture profile for the manufactured snack bars.

	L *	A *	B *	ΔE	a_w_	Hardness (g)	Stickiness (g)
**Control**	76.3 ± 0.87 ^a^	4.17 ± 0.54 ^a^	23.37 ± 0.87 ^a^	--	0.29	2469.3 ± 0.54 ^a^	1205.8 ± 1.02 ^a^
**Bar—BMG**	49.77 ± 1.02 ^b^	10.39 ± 1.05 ^b^	13.62 ± 0.97 ^b^	28.94 ± 1.02 ^a^	0.36	2671.6 ± 1.41 ^b^	1681.4 ± 0.71 ^b^
**Bar—PBD**	36.61 ± 0.62 ^c^	13.29 ± 0.74 ^c^	17.51 ± 1.08 ^c^	41.14 ± 0.81 ^c^	0.34	2089.4 ± 1.37 ^c^	1802.6 ± 0.84 ^c^
**Bar—Mix**	46.24 ± 0.82 ^b^	11.94 ± 0.88 ^b^	12.74 ± 0.69 ^b^	32.82 ± 0.73 ^b^	0.31	2254.9 ± 0.89 ^d^	1354.6 ± 1.08 ^d^

The data are expressed as means ± SD (where *n* = 3). MBG: barley malt grass; PBD: pomegranate by product. L * scale represents the lightness; A * scale represents the redness; B * scale represents the yellowness. The values that appeared with different superscript letters in the same column have significant differences. ΔE was calculated as the square root for the sum of differences between L *, A *, and B * of the treatment and the control.

**Table 6:** The resistance of inoculated bar types for toxigenic fungi contamination.

Days	1	3	5	7	9	11	15
**Inoculated bar**	7.9 × 10^2^	1.6 × 10^3^	4.4 × 10^3^	7.9 × 10^4^	1.4 × 10^5^	5.7 × 10^5^	9.8 × 10^6^
**Bar-BMG**	1.4 × 10^2^	2.8 × 10^2^	5.6 × 10^2^	2.1 × 10^3^	6.8 × 10^3^	1.9 × 10^4^	5.2 × 10^4^
**Bar-PBD**	0.16 × 10^2^	0.41 × 10^2^	0.89 × 10^2^	1.3 × 10^2^	7.3 × 10^2^	2.3 × 10^3^	6.9 × 10^3^
**Bar-Mix**	0.54 × 10^2^	0.96 × 10^2^	1.05 × 10^2^	3.1 × 10^2^	8.9 × 10^2^	3.5 × 10^3^	1.04 × 10^4^

The data of fungal count for exanimate bars are represented as CFU/g bar. MBG: barley malt grass; PBD: pomegranate byproduct. Mix: mean the addition of MBG and PBD at mixing ratio (1:1).

## Data Availability

All the Data regarding this work were represented inside this manuscript.
